# Adaptive training with full-body movements to reduce bradykinesia in persons with Parkinson’s disease: a pilot study

**DOI:** 10.1186/s12984-015-0009-5

**Published:** 2015-02-14

**Authors:** Susanna Summa, Angelo Basteris, Enrico Betti, Vittorio Sanguineti

**Affiliations:** Department of Informatics, Bioengineering, Robotics and Systems Engineering, University of Genoa, Genoa, Italy; School of Computer Science, University of Hertfordshire, Hatfield, UK; Functional Recovery and Rehabilitation Service, Hospital ‘La Colletta’, Arenzano, Italy

**Keywords:** Parkinson’s disease, Bradykinesia, Microsoft kinect

## Abstract

**Background:**

Bradykinesia (slow movements) is a common symptom of Parkinson’s disease (PD) and results in reduced mobility and postural instability. The objective of this study is to develop and demonstrate a technology-assisted exercise protocol that is specifically aimed at reducing bradykinesia.

**Methods:**

Seven persons with PD participated in this study. They were required to perform whole body reaching movements toward targets placed in different directions and at different elevations. Movements were recorded by a Microsoft Kinect movement sensor and used to control a human-like avatar, which was continuously displayed on a screen placed in front of the subjects. After completion of each movement, subjects received a 0-100 score that was inversely proportional to movement time. Target distance in the next movements was automatically adjusted in order to keep the score around a pre-specified target value. In this way, subjects always exercised with the largest movement amplitude they could sustain. The training protocol was organised into blocks of 45 movements toward targets placed in three different directions and at three different elevations (a total of nine targets). Each training session included a finite number of blocks, fitted within a fixed 40 minutes duration. The whole protocol included a total of 10 sessions (approximately two sessions/week).

As primary outcome measure we took the absolute average acceleration. Various aspects of movement performance were taken as secondary outcome measures, namely accuracy (undershoot error), path curvature, movement time, and average speed.

**Results:**

Throughout sessions, we observed an increase of the absolute average acceleration and speed and decreased undershoot error and movement time. Exercise also significantly affected the relationship between target elevation and both speed and acceleration - the improvement was greater at higher elevations.

**Conclusions:**

The device and the protocol were well accepted by subjects and appeared safe and easy to use. Our preliminary results point at a training-induced reduction of bradykinesia.

## Introduction

Bradykinesia (slow movements) is a common symptom in Parkinson’s disease (PD) [1] and has important consequences on daily life activities. As regards the upper limb, it may cause difficulties in dexterous activities such as using work or kitchen tools. It may also contribute to impaired coordination in activities like sport or dressing.

It has been suggested [2] that slow movements are a consequence of a reduced accuracy, which would lead to multiple corrections [3] and therefore to a greater movement time. However, this view is difficult to reconcile with previous observations [4] that movements in PD are characterized by prolonged acceleration phases, not prolonged decelerations as it would have been expected by multiple corrections.

Problems with energy expenditure have often been associated to bradykinesia in PD. Protas et al. [5] and Schenkman et al. [6] suggested that individuals with PD spend about 20% more energy than healthy people during movements, which points at a poor management of energy expenditure in terms of economy of movement. Canning et al. [7] and Stanley et al. [8] showed that, during motor exercise, the attainment of peak aerobic power occurs at a significantly lower exercise level respect to healthy persons, thus indicating poor metabolic efficiency.

Slower movements in PD have also been associated to a reduced muscle strength and to an inability to generate rapid muscle contraction [9]. However, muscle weakness was not consistently observed in all muscles in persons with bradykinesia.

Alteration in sensory processing is another possible explanation. Persons with PD have an abnormal regulation of proprioception; for instance, lack of vision affects the speed/accuracy trade-off more than in controls [10]. However, it is unclear whether these problems arise from altered peripheral feedback or from abnormal central processing [11].

All the above explanations are hard to reconcile with the observation that persons with bradykinesia may indeed perform fast movements, e.g. to escape from a danger (paradoxical kinesia) [12]. Also, persons with bradykinesia can exceed their preferred moving speed while maintaining a movement accuracy comparable to the one of healthy subjects [13]. This suggests that bradykinesia in persons with PD is not a mere compensatory mechanism for impaired motor control or defective sensory processing. Rather, is may be a consequence of an implicit decision to select movements that have a lower energy expenditure or are characterized by lower force levels. Consistent with the emerging view of the role of the basal ganglia as action ‘energizers’ - see [14] for a review - Mazzoni et al. [15] suggested that dopaminergic pathways from the substantia nigra to the striatum may regulate the likelihood of moving at higher speeds.

Rehabilitation may have an important impact in the quality of life of persons with PD. Physical exercise might help to reduce the motor symptoms - especially bradykinesia and balance problems - while keeping the levodopa (LD) dose as low as possible. Also, moderate endurance exercises have been reported to augment the efficacy of LD therapy [16].

A recent review [17] compared the effectiveness of physiotherapy intervention in persons with PD. The study took into consideration a number of common treatments (i.e. general physiotherapy, exercise, treadmill training, cueing, dance, or martial arts). Short-term (i.e. <3 months) benefits of physiotherapy were observed in most outcomes, but were significant only for speed, two- or six-minute walk test, Freezing of Gait questionnaire, Timed Up & Go, Functional Reach Test, Berg Balance Scale, and UPDRS. While many treatments resulted in improved performance, no significant difference was observed between treatments, at least for the outcome measures that were taken into consideration. Recently, a technique originally developed for speech rehabilitation (Lee Silverman’s Voice Therapy, LSVT) has been extended to specifically address motor bradykinesia (Training BIG, later known as LSVT BIG); see [18]. This technique is based on intensive full-body exercise, specifically aimed at increasing the sensory awareness of the widest range of motion that patients can achieve and encouraging the maximum speed. Farley et al. [18] related this technique to the speed-amplitude relation [19] - speed increases with movement amplitude - and observed that training of large amplitude movements involving the whole body induces a modification of this relation - in high-amplitude movements the speed improves more. In a comparative study [20], the LSVT BIG technique resulted in a greater improvement in motor performance with respect to either nordic walking or non-supervised home exercise.

Here we propose a novel approach for reducing bradykinesia, based on virtual reality, exergaming [21] and the low-cost natural user interface Microsoft Kinect. A few studies have tested safety and feasibility of using this device with persons with Parkinson’s disease. Pompeu et al. [22] used a commercial game suite - Microsoft Kinect Adventures™- to engage the player in a variety of mini games that exploit full body motion. Galna et al. [23] used an exercise protocol specifically designed to train dynamic postural control.

Taking inspiration to the LSVT BIG technique, we designed an exercise protocol that relies on whole body reaching movements with different amplitudes and directions, to induce subjects to increase their movement speed and its sensitivity to movement amplitude. Movements were recorded through the Kinect device and displayed on a screen by an animated avatar in a mirror view, which provided subjects with knowledge of their performance. Depending on the measured movement time, an adaptive regulator continuously adjusted the distance of the targets to keep movement time close to a target value established by the therapist. In this way, the exercise was automatically and continuously adapted to the individual’s degree of impairment.

## Materials and methods

### Experimental set-up

The experimental apparatus included a video projector, displaying a virtual reality environment on a 2 m × 2 m screen. Subjects were required to stand in front of the screen, within a 3 m distance. A markerless motion capture sensor (Microsoft Kinect), placed below the screen, recorded the subjects’ full-body movements in 3D space at a 30 Hz sampling rate. The device has a limited accuracy - 1 cm range, see [24] - but allows to reconstruct the trajectories of ‘virtual’ markers in real-time.

Therefore, it can provide participants an immediate, continuous visual feedback of their movements. In our experiment, the reconstructed trajectories of 13 virtual ‘markers’ (one head marker, plus shoulder, elbow, hand, hip, knee, and foot, respectively left and right) were used to animate a ten-segments avatar. Estimates of the markers’ spatial coordinates from the motion sensor data were obtained through the OpenNI (PrimeSense, Tel-Aviv, Israel, [25]) Application Program Interface (API). A specifically developed software application, based on the H3DAPI (SenseGraphics, Sweden, [26]) software environment and Python, was used to implement the task and the experimental protocol (see below).

### Task

**Figure 1:**
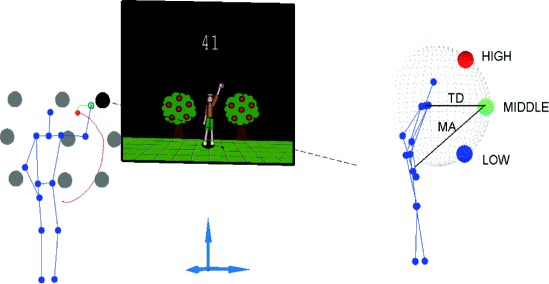
**Target arrangement and visual environment.** Left: The virtual environment consists of an animated avatar, which is continuously showed to the subject, a target point and a numeric score that is displayed after the end of each movement. Each trajectory can be decomposed into an approach (red) and a correction phase (green). The dashed line denotes the line of projection of the target onto the projection center used by the display. Right: The nine targets were placed at a distance TD from the shoulder, at three different elevations: low (blue), middle (green), high (red). For a given target, movement amplitude (MA) denotes the distance of the target from the start hand position.

The proposed exercise protocol involved full-body movements. While standing, subjects were required to reach one of nine targets, presented in random order. The movement was considered as terminated when the hand first entered the target. Therefore, participants were not required to stop their movement when the target was reached. Target positions were defined in terms of a subject-centered reference frame, as points on the surface of two spheres, centered on each shoulder, at elevation angles of −45° (below shoulder, ‘low’), 0° (shoulder level, ‘middle’) and 45° (above shoulder, ‘high’). The targets’ horizontal direction (azimuth) with respect to the ipsilateral shoulder marker was 30° (right), 150° (left) and frontal (intersection of the spheres with the sagittal plane); see Figure 1 for details. The radius of the spheres - i.e. the distance between the targets and the shoulder (target distance, TD) - was initially set to 150% of the subject’s arm length, and was automatically adjusted during the exercise (see below), within the 50-150% range (of arm length). At the beginning of each session, the difficulty level was reset to its initial value. All movements started from a neutral posture in which both arms were extended downward, so that the hands were placed slightly below the pelvis.

A mirror image of the subject was continuously displayed on the screen as an animated avatar, in which the subject’s hands were displayed as ⊘15 cm spheres; see Figure 1 (left). While the subject was in the reference pose, one target (⊘15 cm) appeared on the screen (displayed as either an apple, a star or a bag of money). Subjects were required to reach the target as fast as possible, by using their preferred hand. In other words, subjects were free to choose with which hand to reach the target. In this sense, the task was bilateral. To facilitate reaching, subjects were also allowed to step in all directions.

The task involved movements in three dimensions, but targets were only displayed as projections on the screen placed in front of the subjects. In this way, subjects had a limited information on target location along the ‘depth’ direction. In fact, all points of the projection line connecting the projection center defined by the virtual environment and the 3D position of the virtual target project to the same point of the screen. The only information on ‘depth’ was provided by the size of the displayed target (targets, or body segments, that are further away look smaller when projected). As a consequence, the visual feedback on reaching accuracy was largely two-dimensional (on-screen distance between target and subject’s hand).

The movement was considered completed when either the distance between hand and target was less than the target size, i.e. 15 cm, or movement time was greater than 10 s. After completion of a movement, a 0−100 score was displayed on the screen, calculated as:

(1) $$  \text{score} = 100 \cdot \left \lfloor \frac{1/\text{MT} - 1/\text{MT}_{max}}{1/\text{MT}_{min}-1/\text{MT}_{max}} \right \rfloor  $$

where MT is the total movement time; MT_*max*_ and MT_*min*_ are, respectively, the maximum and minimum durations; and ⌊*x*⌋ is the integer value of *x*. Based on pilot tests with healthy subjects, we set MT_*min*_ and MT_*max*_ to, respectively, 0.5 and 10 s. A zero score was assigned to movements whose duration was greater than MT_*max*_. Movements whose duration was less than MT_*min*_ received a maximum (100) score. We did not explicit tell them that the score was related with MT, but they all realized it after a few epochs. We also provided an audio feedback: (i) an unpleasant sound when a zero score was achieved; (ii) a trumpet sound when score was equal to 100, or (iii) a theme-specific ‘ok’ sound (e.g. a clink if the target was a bag of money) for intermediate score values. In this way, subjects were encouraged to move as fast as possible.

### Exercise protocol

The exercise protocol was organized into epochs, each one corresponding to 5 repetitions of a target set - a sequence of all nine targets, in random order (i.e., 5×9=45 movements per epoch). After each epoch, subjects had to rest (sitting if necessary) for at least 1 min. The therapy protocol consisted of a total of 10 training sessions (2 sessions/week), each lasting 40 minutes. Depending on the individual conditions and thus on individual movement speeds, each session could involve a variable number of epochs. At the beginning of each session, an automatic calibration procedure was carried out to initialize the movement tracking algorithm, to estimate the subject’s arm length and to establish the subject-centered reference frame with respect to which targets were specified. Each phase of this procedure was guided by vocal messages.

We used a Bayesian procedure [27] to automatically adjust the target distance to the individual movement capabilities, on a per target set basis. After completion of a target set (i.e., nine movements), TD was adjusted in order to get the average score in the next target set as close as possible to a pre-specified target value. Specifically, TD was increased if the average score was greater than the target value, and decreased if the average score was smaller. In other words, if a subject could not reach the target fast enough, the next targets were placed closer to the body. If subjects performed well, targets were placed farther away. In this way, subjects always made movements as wide as they could afford but the score, and therefore the average speed, was kept around the specified target score. In our experiments the target score was set to 25/100, corresponding to MT=1.74 s. In summary, subjects were required to maintain a target average performance (quantified by the above duration score) within a pre-specified number of consecutive trials (the ‘target set’) and across different target elevations and movement amplitudes. The adaptive controller automatically adjusted the target distance (i.e., task difficulty) in order to maintain that average score.

### Subjects

The study involved a total of seven subjects with idiopathic PD, see the Table 1 for demographic and clinical information, recruited among the outpatients of the National Health System of the municipality of Genoa, Italy (ASL3 ‘Genovese’).

**Table 1:** **Subjects’ demographic and clinical information**

**Subject**	**Sex**	**Age [y]**	**Disease dur. [y]**	**TUG [s]**	**10MWT [s]**	**UPDRS III (motor) (0-56)**	**H&Y (1-5)**
S1	M	69	13	12*	10*	21	3
S2	M	76	2	12	6	5	2
S3	M	60	5	5	4	5	2
S4	F	65	3	24	12	26	4
S5	M	72	4	38	39*	28	4
S6	F	63	4	6	6	11	1.5
S7	M	67	4	7	8	12	1.5
mean ± SD		67 ± 5	5 ± 4	15 ± 12	12 ± 12	15 ± 10	3 ± 1

M: male, F: female; TUG: Timed ‘Up and Go’ test; 10MWT: 10 Meter Walk Test; UPDRS III (motor): Unified Parkinson’s Disease Rating Scale, part III - motor examination (items 18-31); H&Y: Modified Hoehn and Yahr staging scale. (*) with crutch.

The inclusion criteria were a diagnosis of Parkinson’s disease made by a neurologist and the ability to stand up and make a few steps without a walking aid. Presence of serious psychiatric problems, severe receptive aphasia and inability to perform the Timed ‘Up and Go’ test (TUG) with aids and supervision were taken as exclusion criteria. Presence of early dementia did not in itself constitute an exclusion criterion.

The age of the seven subjects (2M + 5F) was 67±5 years (range 60−76). Disease duration was 5±4 years (range 2−13). We quantified subjects’ impairment through the Unified Parkinson Disease Rating Scale (UPDRS) - part III (motor) - a 0-56 scale (0: normal; 56: maximally impaired) [28] - 15±10 (range: 5−28) and the Modified Hoehn and Yahr (H&Y) staging scale [29,30], a 1-5 scale (1: minimal disability, 5: maximum disability) - 3±1 (range 1.5-4). Before the start of the exercise protocol, the subjects’ performance with the Timed ‘Up and Go’ test (TUG) [31] was 15±12 s (range 5−38 s) and with the 10-Meters-Walk Test (10MWT) [32] was 12±12 s (range: 4−39 s). In the latter test, subjects were instructed to walk as fast as possible. Two subjects (S1 and S3) exhibited an abnormal forward-flexed posture (camptocormia). All subjects were taking medications at the time of testing and were in their ‘ON’ phase during training.

The research conforms to the ethical standards laid down in the 1964 Declaration of Helsinki that protects research subjects. Each subject signed a consent form that conforms to these guidelines.

### Data analysis

The raw recordings of the 3D trajectories of the 13 virtual markers were smoothed with a 4^*th*^ order Savitzky-Golay filter with a 0.96 s time window (corresponding to 29 data samples). The same filter was used to estimate all subsequent time derivatives. The filter parameters correspond to a cut-off frequency of approximately 1.5 Hz. Although relatively low with respect to movement analysis standards, this value is necessary to deal with the low accuracy of the Kinect sensor. The Kinect system uses a reconstruction algorithm to estimate the positions of anatomical points (hand centroid etc.). This reconstruction is not 100% accurate, so that the estimated marker positions tend to fluctuate from one sample to the next. As a consequence, the estimated marker trajectories are more irregular and less smooth than in conventional marker-based motion capture systems [33]. Smoothing reduces this problem. In spite of the limited tracking accuracy of this device [24], the smoothed trajectories still allowed to reliably estimate the main spatio-temporal features of the movement (path, duration, speed).

Movement trajectories can be decomposed into an approach phase, in which subjects reach the target projection line, and a correction phase, in which subjects move along the projection line in order to achieve the target. PD subjects with bradykinesia tend to move slowly and to undershoot the target [34], therefore we expected they had problems with both phases.

In the analysis we only considered the movements that achieved a score greater than zero; the others were rejected. For each movement, we first identified the hand that subjects selected to perform the movement by comparing target distance measurements. We then focused on this hand for all subsequent analysis of each single movement.

We then estimated the movement onset as the instant at which movement speed went above 10% of peak speed. The end of the approach phase was identified as the time when the speed went below this same threshold. Finally, movement end was estimated as the instant at which the distance between the hand and the target was smaller than the target size (i.e. 15 cm).

To assess the effect of exercise, we focused on various aspects of movement performance. In addition to target distance, which is a measure of task difficulty and was automatically adjusted at every target set, for each movement we specifically looked at movement path, movement time and the average absolute acceleration (a measure of movement ‘effort’).

**Movement path** Movements toward a specific target, placed at distance TD from the shoulder, are characterized by a specific Movement Amplitude (MA), defined as the distance between the start position of the hand selected for the movement (i.e. its reference pose) and the target (see Figure 1). This quantity depends on TD but also on target location, thus it is target-dependent. We quantified the movement path in terms of the undershoot error (US), defined as the projection of the endpoint error - difference from target position and final position at the end of the approach phase - over the direction of the target with respect to the start position. As a measure of path curvature we calculated a Linearity index (LI), defined as the relative increase of path length (PL) with respect to the nominal MA: LI=PL/MA−1. A zero LI would correspond to a perfectly straight hand trajectory.

**Movement timing** For each movement we calculated the Movement Time (MT) - which determined the movement score as explained above - defined as the time interval between movement onset and movement end. We also looked at the average speed (AS) for each movement.

**Movement effort** The actual effort that subjects actually devoted to a movement was quantified by taking the average norm of acceleration (AA), calculated as the value of the rectified tangential acceleration, averaged from movement start to movement end (i.e., average of the absolute value of acceleration):

(2) $$ \text{AA}=\frac{1}{MT} \int_{0}^{MT} \left| \frac{dv}{dt} \right| \, dt   $$

where *v*(*t*) is movement speed; see also [15]. In straight-line reaching movements, the average acceleration is proportional to the ratio between path length and the square of movement time, i.e. AA∝PL/MT^2^; see [35,36]. We tentatively assumed that this relation holds in the present task. As a consequence, the score, and thus the reciprocal of movement time, is approximately proportional to the square root of the ratio between the absolute average acceleration and the path length, i.e. $\sqrt {\text {AA}/\text {PL}}$. Hence AA and PL are two major determinants of movement time and therefore of the movement score. Specifically, increasing PL would require an increase of AA in order to keep MT (and thus the movement score) constant.

Since movements toward targets at different elevations have very different amplitudes, we expect that if they are forced to be of equal duration (i.e., equal score), absolute acceleration should also increase with target elevation. In other words, movements toward ‘high’ targets should require more effort to achieve the same score. As the controller regulates the average score and the adjusted target distance is common to all targets, irrespective of their elevation, targets at low elevation - requiring less effort - are expected to achieve a greater-than-average score, whereas targets at high elevation - requiring more effort - will achieve a lower-than-average score.

With training, subjects are expected to improve their overall performance. This means that they should be able to achieve the same target score by reaching more distant targets. Furthermore, for a given target distance, they are expected to put more effort in their movements, i.e they should increase their absolute average acceleration.

#### Statistical analysis

From the recorded hand trajectories, their velocities and their accelerations, we calculated the above indicators for each individual movement. We took the average absolute acceleration as primary outcome measure. All other indicators, which reflect different aspects of task performance, were taken as secondary outcome measures.

To assess the overall effect of exercise on subjects’ performance, for each indicator we ran a 2-way repeated-measures ANOVA, with training (pre- vs post-) and elevation (low, middle, high) as within-subject factors. We compared the trials performed under the most challenging condition, represented by the maximum target distance (150*%* of arm length). For this reason, we took the first epoch of the first session (pre- condition) and the first epoch of the last session (post- condition).

For the indicators that exhibited a significant training and training × elevation effects, we additionally looked at their correlations with disease severity, as measured by the UPDRS - part III and the Modified Hoehn and Yahr staging scale. To do this, for each individual subject and for each indicator we calculated a linear regression over target elevation (low, middle, high), separately for the pre- and post-treatment conditions. We then took: (i) the intercept of the pre-treatment line as pre-treatment performance measure; (ii) the corresponding slope; (iii) the difference in the intercepts of the post- and the pre-treatment lines as a measure of the treatment-related change in performance; and (iv) the difference in the slopes of the post- and pre-treatment lines. For each of the above indicators we took the correlation coefficients with disease severity.

In all cases we took *p*=0.05 as the threshold for statistical significance. We used Matlab (Mathworks, Natick MA) for all data analysis.

## Results

Both the visual environment and the exercise protocol were well accepted by all subjects. Subject S7 exited the study after 5 sessions for health reasons (flu) unrelated to the treatment protocol. This subject was not considered in all further statistical analysis. Although subjects were allowed to step, they very rarely did, likely because they did not feel safe in moving the arm while stepping. In all cases we observed no relevant changes of this behavior as training proceeded. Across sessions, subjects significantly increased (*p*=0.0335; paired samples t-test) the number of completed blocks of trials (epochs) during the (fixed) duration of each session; see Table 2 for details.

**Table 2:** **Number of epochs completed on the first (1) and the last treatment sessions (10)**

**Subject**	**Session 1**	**Session 10**
S1	5	6
S2	7	10
S3	8	9
S4	7	8
S5	7	8
S6	10	10
S7	7	9 ^∗^

Each epoch corresponds to 5 × 9 = 45 movements. (*) Subject S7 exited the study after 5 sessions. The reported number of epochs relates to the last completed session (session 5).

### Regulation of target distance

**Figure 2:**
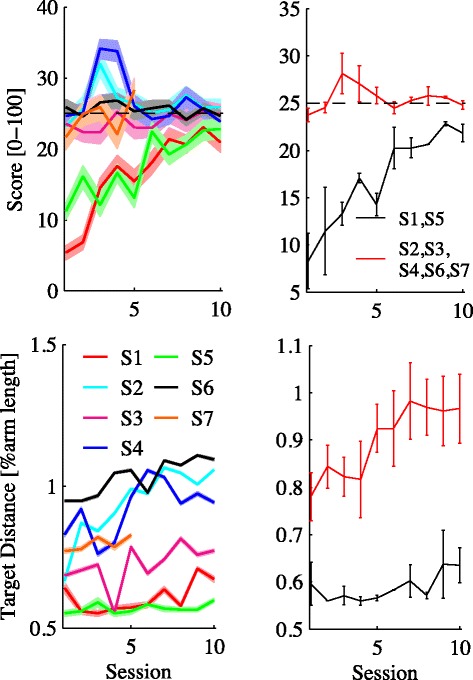
**Temporal evolution of score (top) and TD (bottom).** Left: Individual subjects. Each color represents a different subject. Right: average over subjects (black: S1 and S5; red: all other subjects). Dashed areas and bars denote the standard error (SE).

Based on subjects’ performance (score), task difficulty - i.e. TD - was adaptively regulated ‘as needed’ [27]. In this way, the average score over sessions was expected to gradually get closer to this target value, and a concurrent increase in TD is an indirect indication of improved task performance. Figure 2 (left) shows the temporal evolution of score (top) and TD (bottom), averaged over sessions, for each individual subject. With the exception of subjects S1 and S5 who only approached the target score in the later sessions, all other subjects generally managed to keep their score close to the target value. Across sessions, subjects rapidly reduced the fraction of trials per session in which they got a zero score (target not reached within the timeout interval), from 27 ± 9% to 7 ± 1%. The effect was not significant due to the large between-subject variability.

With the exception of subjects S1 and S5, for which TD remained close to its minimum value (50% of arm length), all other subjects exhibited a gradual TD increase; see Figure 2 (right). Several subjects exhibit a non-monotonic evolution of target distance over sessions. This is because the difficulty level was set to its initial value at the beginning of each session, so that the temporal evolution of TD across sessions exhibits some variability.

### Movement performance

Experimental observations confirmed that subjects generally used a two-step strategy for reaching the targets, consisting of an approach and a correction phase. During the approach phase, subjects reached the line joining the point of view and the actual position in space of the virtual target. All points of this line are projected into the same point on the screen. During the correction phase, subjects moved along this line to achieve the actual 3D target position; see Figure 1 (left).

The results of the 2-way ANOVA are summarized in Table 3.

**Table 3:** **Summary of the results of the 2-way analysis of variance (ns: not significant), for undershoot error (US), linearity index (LI), movement time (MT), average speed (AS) and average absolute acceleration (AA)**

	**Elevation**	**Training**	**Training × Elevation**
US	ns	0.03	ns
LI	ns	ns	ns
MT	ns	0.002	ns
AS	0.008	0.01	0.005
AA	0.02	0.025	0.014

**Figure 3:**
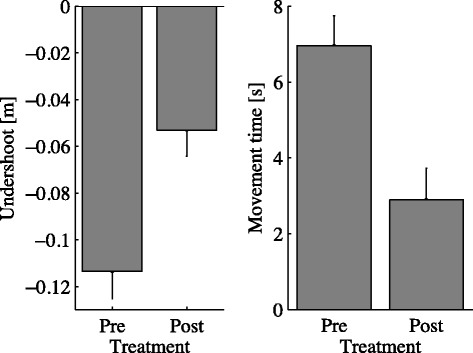
**Effect of training on undershoot error and movement time.** Undershoot Error (left) and Movement Time (rigth) in the first epoch of the beginning (Pre) and the first epoch at the end of the training protocol (Post). Error bars denote the SE.

**Movement Path** During the approach phase, subjects generally tended to undershoot the target, but the magnitude of the effect did not depend on target elevation (non-significant effect of elevation). We observed a significant training effect on the amount of undershoot (*p*=0.03) - undershoot decreases with training. This effect did not depend on target elevation (non-significant interaction between session and elevation); see Table 3 and Figure 3 (left). In contrast, we found no significant changes in path curvature (linearity index, LI) - curvature neither significantly depends on elevation nor significantly decreases with training.

**Figure 4:**
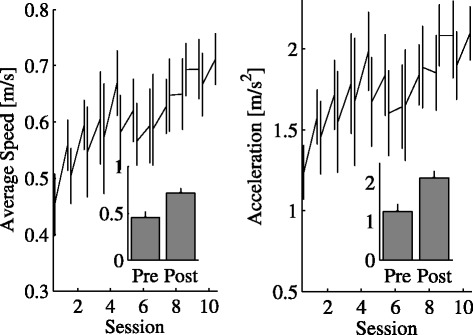
**Temporal evolution and effect of training on average speed and average acceleration.** Average Speed (left) and Average Acceleration (right) averaged across subjects (first and last epoch of each session). Bar graph of Average Speed and Average Acceleration in the first epoch of the beginning (Pre) and the last epoch at the end of the training protocol (Post). Error bars denote the SE.

**Movement Effort** We assessed movement effort in terms of the average absolute acceleration. We found significant training (*p*=0.025) and elevation (*p*=0.02) effects. Figure 4 (right) summarizes the effect of training on movement effort.

**Figure 5:**
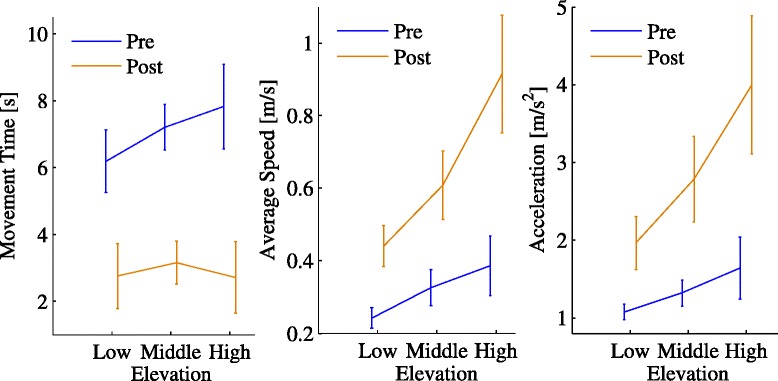
**Interaction between training and target elevation.** Sensitivity of movement time (left), average speed (middle) and average absolute acceleration (right) to target elevation (low, middle, high), respectively at the beginning (Pre, blue line) and at the end of training (Post, orange line). Error bars denote the SE.

In addition, we observed a significant training × elevation effect (*p*=0.014); see Table 3. Figure 5 summarizes this effect.

**Movement timing** We observed a significant decrease (*p*=0.002) of movement time with training; see Figure 3 (right). We did not find significant elevation or elevation × training effects, see Table 3.

As regards average speed, we found a significant elevation effect in the overall movement (*p*=0.008) - speed increases with target elevation. We also found a significant training effect (*p*=0.01); see Table 3. Figure 4 (left) summarizes the effect of training on average speed. We also observed a significant training × elevation interaction (*p*=0.005); see Figure 5.

A look at the relation between MT and elevation - see Figure 5 (left)- suggests that before training MT is significantly greater at high elevation than at low elevation (*p*=0.026, post-hoc comparison with Bonferroni correction). At the end of the training, MT decreases and also becomes less dependent on MA (elevation effect not significant).

### Disease severity

The relation between disease severity of the individual subjects - quantified through the Modified Hoehn and Yahr scale - and the corresponding performance indicators is summarized in Table 4.

**Table 4:** **Correlation of disease severity (Modified Hoehn and Yahr scale, H&Y) with the regression parameters (slope, intercept) of undershoot error (US), movement time (MT), average speed (AS) and average absolute acceleration (AA) with respect to target elevation**

	**US**		**MT**		**AS**		**AA**	
	***R***	**p**	***R***	**p**	***R***	**p**	***R***	**p**
Pre-treatment slope	0.42	0.32	0.36	0.47	-0.82	0.04 ^∗^	-0.74	0.09
Pre-treatment intercept	-0.13	0.80	0.41	0.41	-0.17	0.74	-0.33	0.52
*Δ*slope	-0.54	0.26	0.35	0.49	0.36	0.47	0.23	0.66
*Δ*intercept	0.17	0.73	-0.31	0.54	-0.66	0.15	-0.18	0.73

For each indicator we report the parameter values pre-treatment and the pre-post change. *R* and *p* denote the correlation coefficient and the p-value.

We only found a significant correlation with the pre-treatment movement speed (AS; *R*=−0.82, *p*=0.04) - greater disease severity, less speed. No statistically significant correlations were observed with the UPDRS score.

### Clinical scales

To assess whether the training protocol resulted in modifications of the subjects’ degree of impairment, we performed clinical tests (TUG, 10MWT) before the start and after the end of the training protocol. The TUG score was 15±12 s (range 5−38 s) before training and 16±15 s (range 4−45 s) after training. The 10MWT score, respectively before and after training, was 12±12 s (range: 4−39 s) and 12±13 s (range: 3−37.7 s), see Table 5 for details. We found an improvement in, respectively, the TUG and the 10MWT in 3/6 and 5/6 subjects. However, these effects turned out to be non-significant from the statistical point of view (paired-sample t-test).

**Table 5:** **TUG and 10MWT tests before and after training**

**Subject**	**TUG [s]**		**10MWT [s]**	
	**Before**	**After**	**Before**	**After**
S1	12	14	10	8.7
S2	12	10	6	4.9
S3	5	4	4	3
S4	24	14	12	10
S5	38	45	39	37.7
S6	6	6	6	7
S7	7	NA	8	NA
**mean ± SD**	15 ± 12	16 ± 15	12 ± 12	12 ± 13

Post-training scores for subject S7 are not available (NA) as he did not complete the protocol.

## Discussion

We designed a technology-assisted exercise that specifically aims at increasing movement speed through the repeated practice of large amplitude movements.

Six subjects (out of seven) successfully completed the trial, with the exclusion of S7 who exited the study for reasons unrelated to the treatment. All subjects verbally expressed a high level of acceptance for the treatment and the apparatus. They only reported a difficulty in assessing the 3D location of the targets. This is consistent with a previous study [23] pointing out that, while participants enjoyed the game and could gladly train at home, they exhibited a difficulty to ‘discriminate between different types and orientations of visual objects’.

### Subjects gradually increased movement amplitude

To encourage subjects to exercise at the maximum amplitude they could sustain, we adaptively regulated target distance (and therefore movement amplitude) so that subjects could achieve and maintain a target movement time [27]. This guaranteed both exercising at maximal effort but also safety and motivation (speed and amplitude were maintained within comfortable levels).

Over the training sessions all subjects - see Figure 2 - exhibited a gradual increase of target distance. At the same time, all managed to maintain the movement score (based on movement time) close to the target value of 25/100. The fraction of trials in which subjects got a zero score also rapidly decreased across sessions. We decided to set the same target score for all subjects. For subjects S1 and S5 this was specially challenging, and they only managed to reach it on the final sessions of the training protocol. To all other subjects, the target appeared to be within easy reach, but they still found the task challenging and motivating.

The proposed approach is similar to the LSVT BIG technique, in which subjects are encouraged to practice large amplitude movements through verbal cues by a therapist [18]. In our case, adaptive control of amplitude, time-based reward and the continuous display of the mirror image of the subject, of his/her movements and of the targets plays a similar role of the verbal cues used by [18], as a way to promote subjects’ awareness of the amplitude of their movements. Sensory awareness of movement magnitude is related to the integration of proprioception and vision, which is another essential aspect of the LSVT BIG technique.

### Subjects become faster and more accurate

With training, we expected subjects to gradually improve both precision and speed of their movements.

As regards precision, irrespective of target elevation subjects generally tended to undershoot the target. This is a well-documented symptom - hypometria - that has often been related to bradykinesia [2,37]. Specifically, bradykinesia may in part result from a reduced endpoint accuracy. Sheridan and Flowers [38] hypothesized that in order to maintain accuracy within acceptable limits, PD patients are forced to increase the duration of their movements. However, we suspect that in the present experiment the observed undershoot may be at least partly a consequence of a parsimonious strategy (i.e. ‘stopping early’) to deal with the lack of depth information. In fact, we ran few trials with healthy subjects and they reported similar problems (data not shown). Nevertheless, with training we indeed observed a significant decrease of the undershoot error; see Figure 3 (top).

We also observed a significant decrease in the movement time - see Figure 3 (top) - and a corresponding increase in movement speed and in absolute acceleration - subjects tend to move faster and to put more effort in their movements increasing also their accuracy; see Figure 3 (bottom). A further, more indirect indication that subjects move faster is represented by the significant increase of the number of movements that subjects managed to complete within each 40-min training session.

Finally, we looked at the relation between the amount of improvement (in motor performance, in motor motivation) and the initial degree of impairment, as measured by the Modified Hoehn and Yahr score and the UPDRS-III scale. We found a weak but significant negative correlation between disease severity and the pre-treatment speed - more severely impaired subjects initially make slower movements. In contrast, no significant relationship was observed between disease severity and performance improvement. These results suggest a simple relation between task-related performance measure and the overall degree of impairment. However, they should be taken cautiously given the small number of subjects that are far from representative of the general PD population.

### Reduced bradykinesia or task familiarization?

An improved speed and accuracy of the movement may result from either a true reduction of the bradykinesia symptoms, or mere familiarization with the task.

As mentioned in the Introduction, bradykinesia has been associated to either a difficulty in selecting movements that require greater levels of energy expenditure [15,39,40] or an insensitivity to rewarding outcomes [41]. Formulations based on optimal control - e.g. [40] - emphasize that movements are the result of a trade-off between reward and effort. Response vigor - the bias toward selecting high-speed movements - reflects this trade-off. The notion that the latter is mediated by the basal ganglia has found some empirical confirmation [14,42].

Vigor is difficult to quantify empirically [15]. Some studies have been looking at the observation that movement speed increases with movement amplitude - the amplitude-speed effect, see [18]. This relation has been reported in reaching, in walking, in handwriting and in eye movements. For instance, Choi et al. [43] analyzed saccades of various amplitudes and looked at the relationship between amplitude and speed, and how it depends on the subjects’ degree of impulsivity, defined in terms of how long they are willing to wait for a rewarding outcome. Their main finding was that subjects’ impulsivity correlated with the slope of the saccade’s amplitude-speed relationship. In other studies [44] this effect was quantified in terms of the relationship between movement amplitude and the average acceleration, taken as a measure of effort. These authors reported that the handwriting movements of PD subjects have an abnormal stroke size - acceleration dependence.

Taken together, the above studies suggest that the slope of the amplitude-speed or amplitude-acceleration dependence can be taken as a measure of vigor. In the present study we looked at the slopes of both the amplitude-acceleration and the amplitude-speed relations. We observed a significant effect of elevation (or, equivalently, amplitude) in the average absolute acceleration, which more directly reflects energy expenditure; see Table 3 and Figure 5. A similar effect was observed in the average speed - training led to an increase of the slope of the amplitude-speed relation.

However, one problem with this interpretation is that familiarization with the task would result, by itself, in a generalized increase of movement speed, while not necessarily implying a vigor change.

As regards the amplitude-absolute acceleration relationship, Rigoux et al. [40]’s model predicts that low vigor - i.e. a greater subjective importance given to movement effort - implies a greater sensitivity of MT to MA. To further explore this point, we looked at the empirical relation between MT, elevation (i.e. MA) and training; see Figure 5 (left). We found that before training MT is significantly greater at high elevation than at low elevation. At the end of the training MT not only decreases, but also becomes less dependent on MA (elevation effect no longer significant). Similar findings were reported by van Gemmert et al. [44] in the context of handwriting. They specifically looked at the relationship between the size and the duration of elementary movements (stroke), in healthy subjects and in persons with PD.

Hence, our data exhibit an effect that is consistent with an increased vigor [40]. However, familiarization with the task would lead to a reduced MT in ways that are similar to those induced by vigor change, so that these aspects would be difficult to distinguish. Therefore, a slope increase in the AA vs MA relation may be at least in part a consequence of familiarization with the task. Similar considerations apply to the AS vs MA relation.

In summary, our observed training-induced changes in both the amplitude-speed or the amplitude-acceleration relations are consistent with an increased vigor but are not conclusive in distinguishing between task familiarization and vigor change.

### Toward clinical application

Although our findings are far from conclusive and expect confirmation by a larger study, they nevertheless suggest a training-induced improvement of the bradykinesia symptoms.

We observed a modest improvement in some subjects in a variety of clinical scales, but these changes were not statistically significant. In contrast, Ebersbach et al. [20] delivered 1-hour treatment sessions, 4 sessions/week for 4 weeks (a total of 16 hours of treatment) and found a clinically significant reduction of the UPDRS-III score. A lower reduction was observed after a shorter duration (2 weeks) version of the same LSVT protocol [45] (a total of 8 hours of treatment). After a Kinect-based training protocol consisting of fourteen 60-minute sessions with the Kinect Adventure game suite (a total of 14 hours of treatment), Pompeu et al. [22] also reported an improvement in activity (balance and gait) and participation (quality of life).

It should be noted that our subjects only made 40-minutes treatment sessions, 2 sessions/week for 5 weeks (a total of 6.6 hours of treatment), which is a far lower dose than [20,22] but is similar to [45]. The better outcome of the latter may depend on the different intensity (similar treatment doses administered in half the time) and/or the behavioral training provided in addition to the large amplitude exercise. In all cases we found no evidence of plateau effects in the temporal evolution of performance indicators in Figure 4, which suggests that additional exercise might have resulted in even more improvement.

Another limitation of our proposed approach with respect to the LSVT BIG technique is that, although we provided several forms of feedback on task performance, we did not explicitly stimulate subjects’ motivation and we did not explicitly promote transfer of the improved performance to activities of daily living. Using a tangible (monetary) reward and/or directly measuring enjoyment, and possibly modulating them during training might further improve the outcome.

## Conclusions

We have explored the potential of the Microsoft Kinect by focusing on two specific symptoms of Parkinson’s disease, namely bradykinesia and hypokinesia.

The rationale underlying the study is that bradykinesia can be mitigated by repeated exercise that specifically focuses on high-amplitude movements [18,20].

Although preliminar, our results point at a training-induced reduction of bradykinesia. However, we cannot conclude on whether the observed outcome is the mere effect of familiarization with the task or is a consequence of an increased vigor. Proper discrimination between these two effects is indeed an open issue, which we leave to future developments. To address this, one could possibly focus on more automatic motor activities (e.g. handwriting, speech, etc), for which a familiarization effect can be ruled out, or on comparing the effects of training with a baseline (e.g. healthy subjects, or PD subjects ON vs OFF medication). Another possibility is to use computational models that explicitly address learning and vigor change, to estimate learning-related and vigor change-related contributions to the observed changes of performance. The same arguments on the difficulty of distinguishing between performance improvements related to familiarization and those related to vigor also apply to assessing bradykinesia through clinical scales, none of which specifically address or vigor.

More in general, we wanted to explore the potential of natural user interfaces as rehabilitation devices. Natural interfaces are appealing because subjects can freely move and are not required to wear sensors or markers. This makes their use more intuitive and more comfortable specially for older users. In fact as in other reports the device was well accepted by our subjects and appeared safe and easy to use. In the context of rehabilitation they are increasingly used in conjunction with off-the-shelf video games [22], but they also allow to design exercises that target specific types of impairment [23]. One secondary aspect is the low cost, which makes this treatment particularly affordable by rehabilitation centers and even individual users. Taken together, these aspects suggest that the proposed treatment may be suitable for training with little or no supervision by a therapist, possibly in domestic environments.
